# Isolation of *TSCD11* Gene for Early Chloroplast Development under High Temperature in Rice

**DOI:** 10.1186/s12284-020-00411-6

**Published:** 2020-07-17

**Authors:** Guonan Fang, Shenglong Yang, Banpu Ruan, Chaolei Liu, Anpeng Zhang, Hongzhen Jiang, Shilin Ding, Biao Tian, Yu Zhang, Noushin Jahan, Li Zhu, Guangheng Zhang, Guojun Dong, Qiang Zhang, Dali Zeng, Longbiao Guo, Zhenyu Gao, Qian Qian

**Affiliations:** State Key Laboratory of Rice Biology, China National Rice Research Institute, Hangzhou, 310006 China

**Keywords:** *TSCD11*, Seryl-tRNA synthetase, Temperature-sensitive, Chloroplast development, Rice

## Abstract

**Background:**

Chloroplasts are essential for photosynthesis and play key roles in plant development. High temperature affects structure of chloroplasts and metabolism in plants. The seryl-tRNA synthetase plays an important role in translation of proteins. Although seryl-tRNA synthetase has been widely studied in microbes and animals, few studies have reported about its role in chloroplast development under high temperature in rice.

**Results:**

In this study, we isolated a novel *temperature*-*sensitive chlorophyll*-*deficient 11* (*tscd11*) mutant by ethyl methane sulfonate (EMS) mutagenesis of *japonica* variety Wuyujing7. The *tscd11* mutant developed albino leaves at the 3-leaf stage under high temperature (35 °C), but had normal green leaves under low temperature (25 °C). Consistent with the albino phenotype, impaired chloroplasts, decreased chlorophyll content and increased ROS accumulation were found in the *tscd11* mutant at 35 °C. Fine mapping and DNA sequencing of *tscd11* revealed a missense mutation (G to A) in the eighth exon of *LOC_Os11g39670* resulted in amino acid change (Glu_374_ to Lys_374_). The *TSCD11* gene encodes a seryl-tRNA synthetase localized to chloroplast. Complementation test confirmed that the point mutation in *TSCD11* is responsible for the phenotype of *tscd11*. *TSCD11* is highly expressed in leaves. Compared with the wild type (WT), mutation in *TSCD11* led to significant alteration in expression levels of genes associated with chlorophyll biosynthesis, photosynthesis and chloroplast development under high temperature.

**Conclusions:**

*TSCD11*, encoding a seryl-tRNA synthetase localized to chloroplast, is vital to early chloroplast development at high temperature in rice, which help to further study on the molecular mechanism of chloroplast development under high temperature.

## Background

Global warming poses many severe challenges to human life, for example, by dramatically reducing crop yields (Battisti and Naylor 2009; Bohra-Mishra et al. 2014; Lobell et al. 2011; Peng et al. 2004). In order to adapt to the harsh environment, plants have evolved multiple tolerance strategies (Bita and Gerats 2013). Under favorable temperature conditions, plants can perform photosynthesis and grow well. While facing extremely unfavorable temperature conditions, plants acclimate to stresses by fine-tuning gene expression in response to environments. Genomic and genetic evidence suggests that various proteins participate in plant thermal response (Jacob et al. 2017; Kumar and Wigge 2010).

Chloroplasts have the characteristics of distinguishing plant cells from animal cells. They are the organs necessary for photosynthesis of higher plants and are also responsible for biosynthesis and storage of various metabolites (Jensen and Leister 2014). The plastid and nuclear genome together control the formation of chloroplast from proplastid, this procedure is affected by environmental conditions such as light and temperature (López-Juez 2007; Mullet 1993). More and more evidence have revealed that chloroplast biogenesis is highly regulated. Photosystem II (PSII) is the least heat-resistant component of the chloroplast thylakoid membrane, and heat stress induces dysfunction of the oxygen releasing complex by imposing sustained oxidative damage to the chloroplast (Allakhverdiev et al. 2008; Yang et al. 2019). In addition, thermal stress usually leads to the destruction of the overall structural integrity of chloroplast thylakoid membranes, especially destacking of thylakoid membranes, which eventually hampers chloroplast development (Semenova 2004; Yamamoto et al. 2008). Chloroplast is a semi-autonomous organelle that encodes about 100 genes (Delannoy et al. 2009). Mutations in these genes often result in a large number of macroscopic leaf-color mutants. Some genes have been cloned in rice so far including *Mg-chelatase H subunit* (*OsCHLH*), *Virescent 1* (*V1*), *Chlorophyll a Oxygenase* (*OsCAO1*)*, Yellow Leaf and Early Flowering* (*YE1*)*, Heat-stress Sensitive Albino 1* (*HSA1*)*, Virescent 2* (*V2*), *Yellow-green Leaf 1* (*YGL1*)*, Virescent 3* (*V3)*, *Stripe1* (*ST1*) and *Thermo-sensitive Chlorophyll-deficient Mutant 5* (*TCM5*) (Jung et al. 2003; Kusumi et al. 2011; Lee et al. 2005; Peng et al. 2019; Qiu et al. 2017; Sugimoto et al. 2007; Wu et al. 2007; Yoo et al. 2009; Zheng et al. 2016). Only minority of them are high temperature sensitive, including *HSA1* and *TCM5,* which are considered to be ideal materials for studying the mechanisms underlie the response to heat stress during chloroplast development.

Aminoacyl-tRNA synthetases (AARSs) are essential enzymes for protein synthesis. The classical function of AARSs are to catalyze the connection of specific amino acids to the corresponding tRNA to form aminoacyl tRNA, then according to the principle that the anti-codon of the tRNA matches the codon of the mRNA, the amino acids are sequentially connected on the ribosome to synthesize the protein. This reaction is the first step in protein synthesis (Banerjee et al. 2011; Pak et al. 2018). In plants, protein synthesis occurs in the cytoplasm, mitochondria and chloroplasts (Schimmel and Soll 1979). Although AARSs are highly conserved enzymes, they show diverse functions, such as splicing of RNA, participation in immune response and tRNA proofreading (Guo et al. 2010; Martinis et al. 1999; Szymański et al. 2000). Because amide-tRNA synthetase is critical in cells, mutants of AARSs are rarely reported. The *edd1* mutant with inactivated glycyl-tRNA synthetase in Arabidopsis, showed embryo-defective in development (Uwer et al. 1998). In Arabidopsis, the *twn2* mutant had altered expression of valyl-tRNA synthetase and exhibited suspensor-derived polyembryony (Zhang and Somerville 1997). In rice, the *wp1* mutant with a signal-nucleotide substitution in the gene coding for val-tRNA synthetase, showed white stripe and albino phenotypes at the seedling stage, and white panicle phenotype at the heading stage, indicating its role in early chloroplast development in rice (Wang et al. 2016). Few of cloned AARSs genes have been reported to affect chloroplast development under high temperature. The sertyl-tRNA synthetase has been reported frequently in microbes and animals, however, rarely in plants. In order to study the cellular function of plant sertyl-tRNA synthetase, virus induced gene silencing (VIGS) was utilized to generate mutants with reduced expression of sertyl-tRNA synthetase in *Nicotiana benthamiana*, and a severe yellowing leaf phenotype was found. The number and size of chloroplasts, and chlorophyll content were also dramatically reduced in the mutants (Kim et al. 2005). The result indicated that sertyl-tRNA synthetase may play a key role in chloroplast development.

Although the seryl-tRNA synthetase has been widely studied in microbes and animals, few have been reported about its roles in chloroplast development under high temperature in rice. In this study, we identified a novel *tscd11* mutant with sensitivity to high temperature. The mutant exhibited albino leaves phenotype at the 3-leaf stage at 35 °C, whereas had normal green leaves at 25 °C. Map-based cloning of the *TSCD11* gene revealed it encoding seryl-tRNA synthetase, which is vital to chloroplast development under high temperature.

## Main Text

## Results

### Phenotypes of the *tscd11* Mutant in Different Environmental Temperatures

**Fig. 1:**
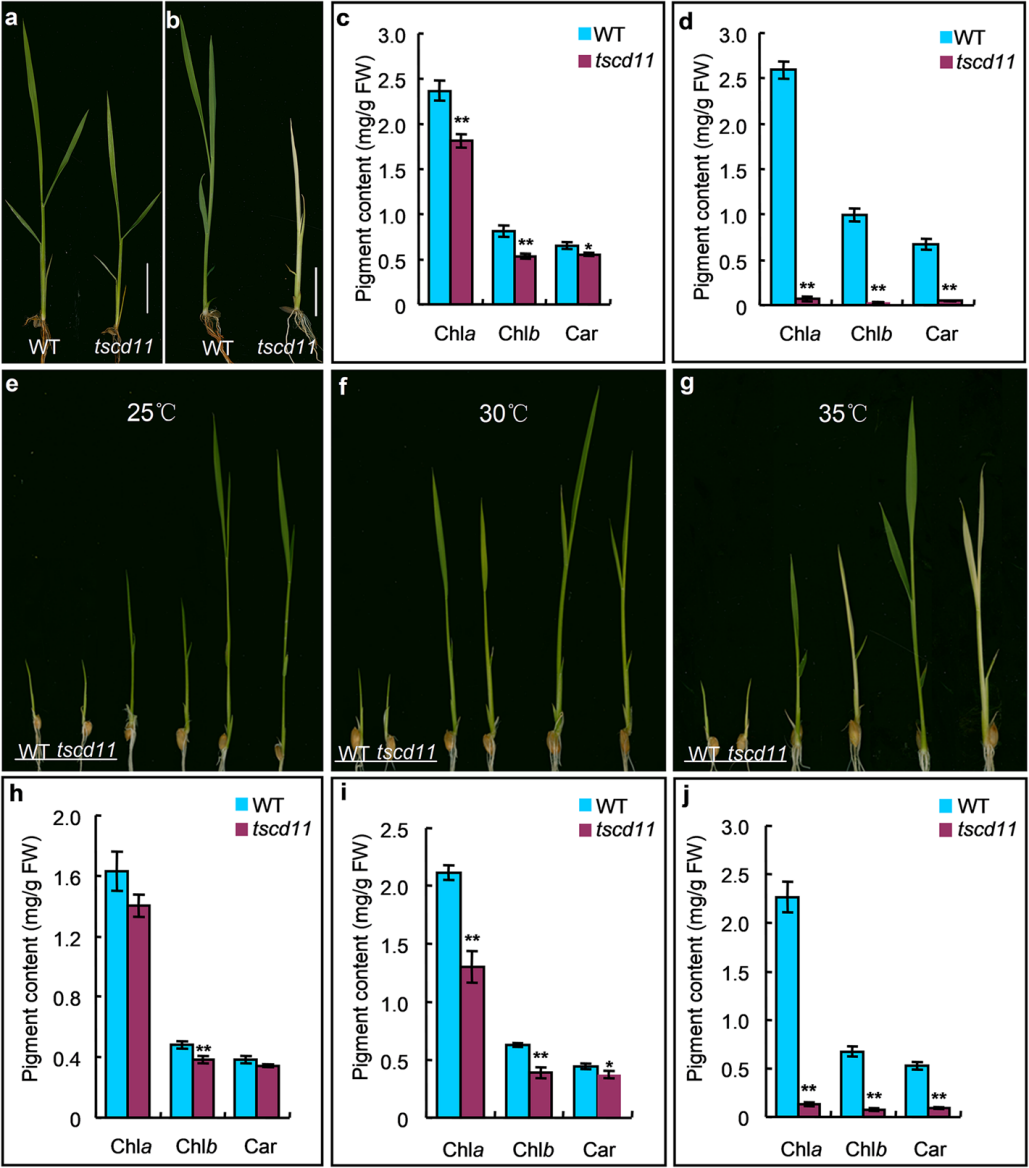
Phenotypes and pigment contents comparison between wild-type and *tscd11* plants. **a-b** Morphology of wild-type and *tscd11* plants at the seedling stage sown in May and June, respectively, in the paddy field (bar = 2 cm).**c-d** pigment contents of WT and *tscd11* at the seeding stage in May and June, respectively. **e-g** 1-, 2-, 3-leaf stage seedlings of the WT and *tscd11* mutant grown at 25 °C, 30 °C and 35 °C, respectively. **h-j** Pigment contents of 3-leaf stage of the wild-type and *tscd11* plants grown at 25 °C, 30 °C and 35 °C, resepectively. Chl *a,* chlorphyll *a*; Chl *b,* chlorphyll *b*; Car, carotenoids. Data represent mean ± SD (*n* = 3). * *p* < 0.05, ** *p* < 0.01 (Student’s *t*-test)

At the seedling stage, the *tscd11* mutant plants exhibited different phenotypes with different sowing date in the paddy field. When the *tscd11* mutant plants were sown in May, they developed white-striped leaves, and their contents of chlorophyll *a* (Chl *a*), chlorophyll *b* (Chl *b*) and carotenoid (Car) were decreased by 23.2%, 34.6% and 14.8% compared to the wild-type plants, respectively (Fig. 1a, c). The leaves exhibited albino in the June sowing mutant plants, and their contents of Chl *a*, Chl *b* and Car in *tscd11* were only 2.7%, 3.2% and 7.6% that of the wild-type plants, respectively (Fig. 1b, d). To test whether the phenotypic difference of *tscd11* is affected by temperature, plants of the WT and *tscd11* were treated with different temperatures in growth chambers. The *tscd11* mutant showed almost the same phenotype as the WT at 25 °C, with similar Chl *a* and Car contents (Fig. 1e, h). The *tscd11* mutant exhibited yellowish green phenotype at 30 °C (Fig. 1f), with the contents of chlorophyll (Chl) decreased accordingly compared with the WT (Fig. 1i). At 35 °C, the *tscd11* mutant displayed albino phenotype at the 3-leaf stage (Fig. 1g), and could not survive until the 4–5 leaf stage. The Chl *a*, Chl *b* and Car were only 5.9%, 12.0% and 18.0% contents of the WT (Fig. 1j). Furthermore, as shown in Fig. 1g, the *tscd11* mutant developed albino leaves from the 2-leaf stage at 35 °C. Therefore, we performed temperature shift experiments at the 2-leaf stage. As shown in Fig. S1f, the new third leaf of the *tscd11* mutant showed albino phenotype when shifted from 25 °C to 35 °C at the 3-leaf stage, and the contents of Chl *a*, Chl *b* and Car in *tscd11* were only 9.5%, 16.9% and 20.6% that of the WT, respectively (Additional file 1: Fig. S1f, j). While the new third leaf of *tscd11* exhibited white-stripe leaf phenotype when shifted from 35 °C to 25 °C at the 3-leaf stage. And although the contents of Chl *a*, Chl *b* and Car in *tscd11* decreased significantly compared to the WT from 35 °C to 25 °C, the pigment contents of *tscd11* were significantly higher from 35 °C to 25 °C than those at 35 °C (Additional file 1: Fig. S1g, h, k, l; Additional file 2: Table S1). Therefore, pigment synthesis in the *tscd11* mutant is temperature dependent. Moreover, differences in pigment contents became smaller in the paddy field as climate temperature decreased from summer till fall (Additional file 1: Fig. S2) in Hangzhou of China. These results illustrate that the *tscd11* mutant is sensitive to high temperature.

In addition, agronomic traits of the WT and *tscd11* plants were investigated in the paddy field. Compared with the WT, plant height, panicle length, seed-setting rate and 1000-grain weight were significantly decreased in *tscd11*, indicating the mutation also inhibits plant growth (Additional file 2: Table S2).

### Chloroplast Development Is Hampered in the *tscd11* Mutant under High Temperature

**Fig. 2:**
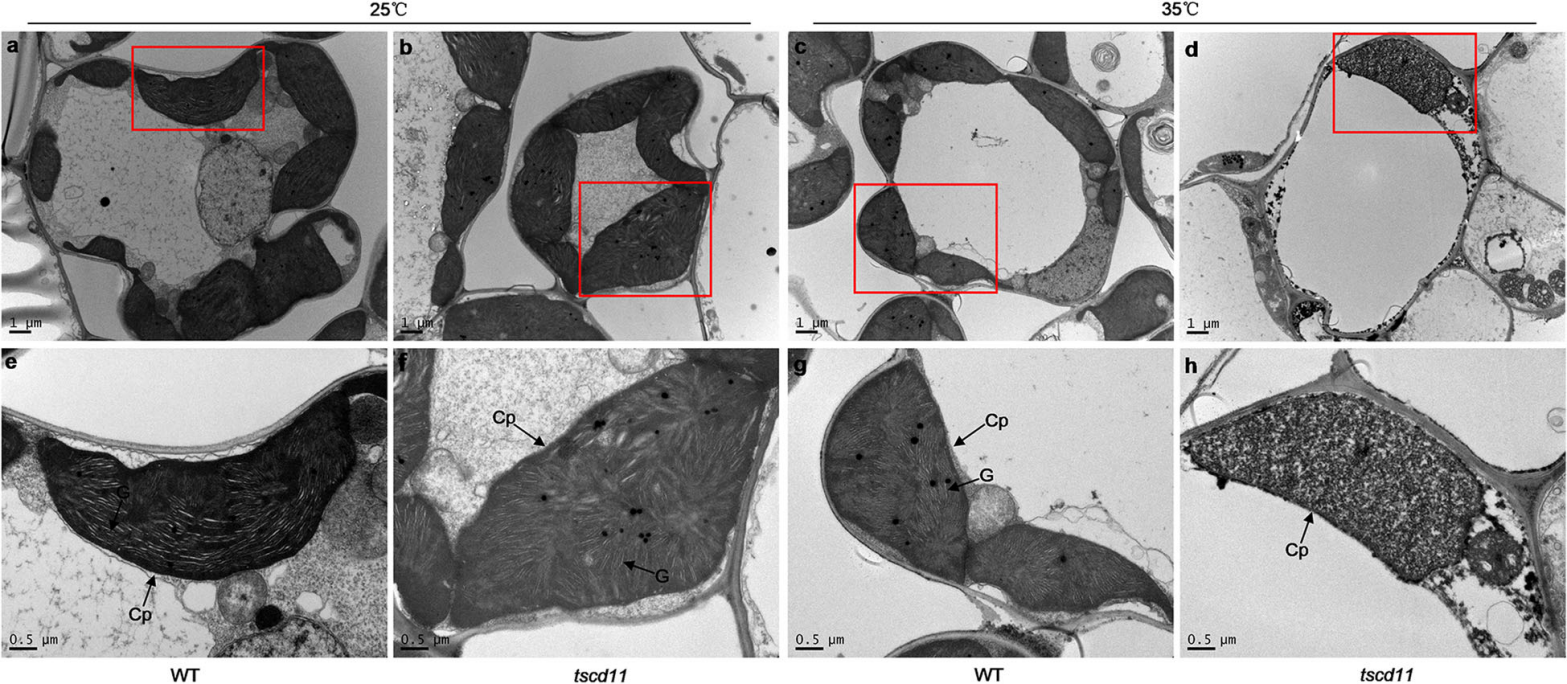
Transmission electron micrographs of chloroplasts in WT and the *tscd11* mutant. **a, b** Chloroplasts structure from WT and *tscd11* at 25 °C. **c**, **d** Chloroplasts structure from WT and *tscd11* at 35 °C. **e-h** are enlargements of **a-d**, respectively. CP, chloroplast; G, grana lamella stacks

The chloroplast ultra-structure of the WT and the *tscd11* mutant were observed at the 3-leaf stage by transmission electron microscopy (TEM). The chloroplast ultra-structure of *tscd11* changed little at 25 °C (Fig. 2a-b, e-f). By contrast, chloroplasts were observed impaired without stacked grana or stromal thylakoids in *tscd11* compared with the WT at 35 °C (Fig. 2c, d, g-h). These results suggest that mutation hampered chloroplast development in the *tscd11* mutant under high temperature.

### More ROS Accumulation in the *tscd11* Mutant under High Temperature

Increased accumulation of reactive oxygen species (ROS) usually occurs in weak plants under high temperature (Chen et al. 2017; Chen et al. 2018). To verify this hypothesis, we performed 3, 3′-diaminobenzidine (DAB) staining and nitro blue tetrazolium (NBT) staining tests to detect hydrogen peroxide (H_2_O_2_) and superoxide anions (O_2_^−^), two indicators of ROS. Numerous H_2_O_2_ and O_2_^−^ accumulated in the albino leaves of *tscd11* grown at 35 °C (Additional file 1: Fig. S3a, b, f, g). We then measured contents of senescence-related substances, H_2_O_2_ and malondialdehyde (MDA), and catalase (CAT) activity at 25 °C and 35 °C. Contents of H_2_O_2_ and MDA increased, and CAT activity decreased in *tscd11* compared to the WT at 25 °C, however, the differences did not reach significant levels (Additional file 1: Fig. S3c-e). In contrast, contents of H_2_O_2_ and MDA significantly increased, and CAT activity significantly decreased in *tscd11* compared to the WT at 35 °C (Additional file 1: Fig. S3h-j). These results were consistent with the staining results with DAB and NBT, indicating that the mutation results in more ROS accumulation in the *tscd11* mutant under high temperature stress. Due to significant differences in peroxide levels between the WT and the *tscd11* mutant at high temperature, we measured transcriptional expression of some senescence related genes including *WRKY transcription factor* (*WRKY72*), *Osh36*, *Stay Green Rice* (*SGR*), ROS-responsive genes *WRKY transcription factor* (*WRKY24*, *WRKY70*), and detoxification related genes *Alternative Oxidase* (*AOX1a*, *AOX1b)*, *Ascorbate Peroxidase* (*APX1*), *Catalase* (*CATB*) and *Superoxide Dismutase* (*SODA1*, *SODB*) in the *tscd11* and wild-type plants. At 25 °C, the transcript levels of most senescence, ROS-responsive and detoxification related genes showed no significant difference between the wild-type and *tscd11* plants (Additional file 1: Fig. S3k). While at 35 °C, the transcript levels of all tested genes were significantly higher in *tscd11* plants than those in the wild-type plants (Additional file 1: Fig. S3l). To examine cell death in *tscd11* at molecular level, the top leaves from the wild-type and *tscd11* plants at the 3-leaf stage were subjected to the terminal deoxynucleotidyl transferase-mediated dUTP nick-end labeling (TUNEL) assay according to the method of Huang et al. (2007). The same leaf sections were synchronously stained with propidium iodide (PI) to indicate nuclei (red) in each section. Fewer nuclei were TUNEL positive in the WT grown at 25 °C or 35 °C and *tscd11* grown at 25 °C; On the contrary, more nuclei in *tscd11* showed TUNEL positive compared with the WT (Additional file 1: Fig. S4). The result reveals that the mutation affects DNA stability in the *tscd11* mutant under high temperature.

### Genetic Analysis and Map-Based Cloning of *TSCD11*

Genetic analysis of reciprocal crosses were performed between the *tscd11* mutant and *japonica* variety Wuyujing7 or *indica* variety 93–11. Leaves had a normal, green appearance in all F_1_ plants. Among the F_2_ population, the segregation ratio of normal green to albino was in accordance with 3:1 (Additional file 2: Table S3). These results indicate that the *tscd11* phenotype is controlled by a single recessive nuclear gene.

**Fig. 3:**
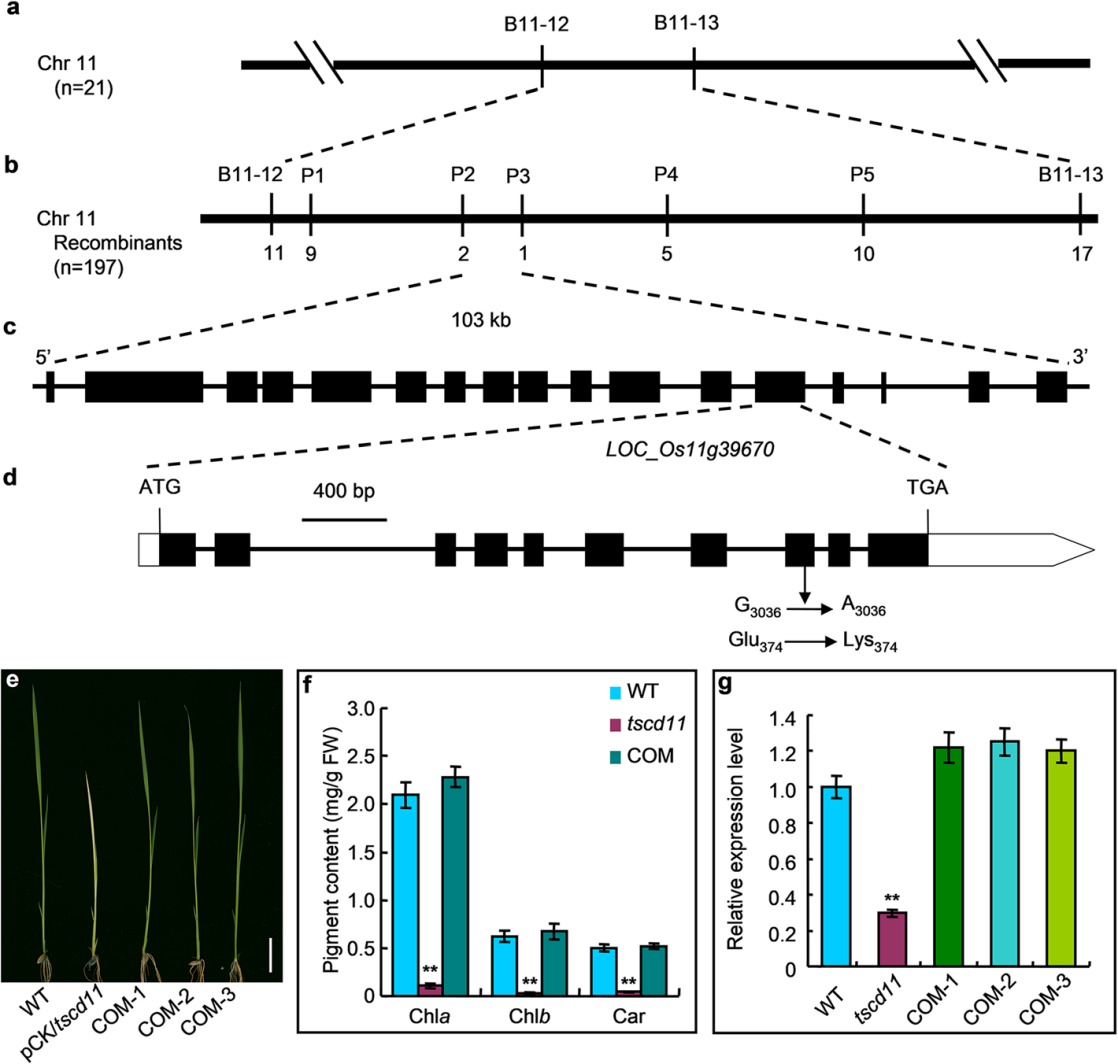
Map-based cloning of *TSCD11* and transgenic complementation of the *tscd11* mutant. **a**, **b** The *TSCD11* locus was initially mapped on chromosome 11. **c**, Fine mapping the *TSCD11* locus. **d**, *TSCD11* gene structure and mutation site of *TSCD11*. **e**, phenotypes of wild-type, *tscd11* with pCMBIA1300 (pCK/*tscd11*) and three independent *tscd11* complementation plants (COM-1, 2 and 3) at 35 °C (bar = 2 cm). **f**, pigment contents of wild-type, *tscd11* and complementation plants **g**, Relative transcript levels of *TSCD11* in wild-type, *tscd11* and complementation plants at the 3-leaf stage at 35 °C. The rice *Histone* gene was used as an internal control. The transcript level of *TSCD11* in the WT at 35 °C was set to 1.0. Data represent the mean ± SD (n = 3). ** *p* < 0.01 (Student’s *t*-test)

For fine mapping of the *TSCD11* locus, we generated a F_2_ mapping population from the cross between the *tscd11* mutant and *indica* variety 93–11. Using 21 F_2_ mutant individuals, we mapped *TSCD11* between STS markers B11–12 and B11–13 on chromosome 11 (Fig. 3a). To further fine map the *TSCD11*, we designed new insertion/deletion (INDEL) markers between B11–12 and B11–13 based on sequence differences between 93 and 11 and Nipponbare. With 197 F_2_ mutant individuals, the *TSCD11* locus was finally narrowed to a 103-kb region between INDEL markers P2 and P3 (Fig. 3b). The region includes 17 predicted ORFs (http://rice.plantbiology.msu.edu/index.shtml; Fig. 3c). Sequencing of all annotated genes in target region found a single nucleotide mutation (G → A) at 3036 bp from the start codon ATG in *LOC_Os11g39670*, which resulted in an amino acid substitution of lysine for glutamic acid (Fig. 3d).

To verify that the single nucleotide mutation of *TSCD11* is responsible for the *tscd11* mutant phenotype, we constructed a complementation vector with a wild-type genomic fragment containing the entire coding region of *TSCD11*, along with 1999 bp of upstream promoter sequence and 846 bp of downstream sequence. The fragment was inserted into the binary vector pCAMBIA1300, and the construct pCAMBIA1300-*TSCD11* was introduced into the *tscd11* mutant by *Agrobacterium*-mediated transformation. As expected, all 26 independent transgenic plants with pCAMBIA1300-*TSCD11* displayed WT phenotype under high temperature, whereas all 5 independent lines transformed with the empty vector maintained *tscd11* mutant phenotype (Fig. 3e). Consistently, contents of Chl and Car, together with expression levels of *TSCD11* in the wild-type plants and all complementation plants were significantly higher than those of *tscd11* plants under high temperature (Fig. 3f, g). These results confirm that *LOC_Os11g39670* is *TSCD11*.

### Sequence and Phylogenetic Analysis of TSCD11

**Fig. 4:**
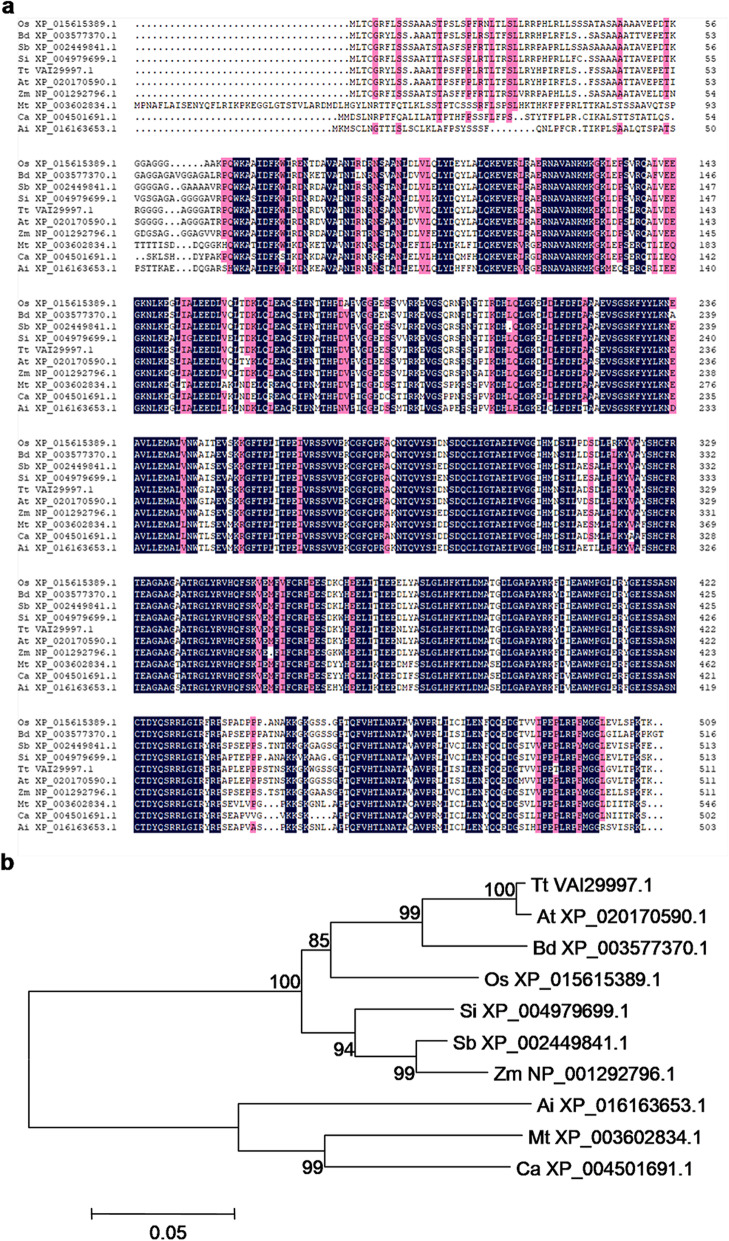
Phylogenic analysis of TSCD11. **a**, Amino acid sequence alignment of 10 types of TSCD11 homologs. Fully or partially conserved amino acids are shaded blue and pink, respectively. **b**, Phylogenic tree of TSCD11 and its homologs. Protein sequences are *Oryza sativa* (Os XP_015615389.1), *Brachypodium distachyon* (Bd XP_003577370.1), *Sorghum bicolor* (Sb XP_002449841.1), *Setaria italica* (Si XP_004979699.1), *Triticum turgidum* (Tt VAI29997.1), *Aegilops tauschii* (At XP_020170590.1), *Zea mays* (Zm NP_001292796.1), *Medicago truncatula* (Mt XP_003602834.1), *Cicer arietinum* (Ca XP_004501691.1), *Arachis ipaensis* (Ai XP_016163653.1). The phylogenetic tree was constructed by MEGA version 6.0 software based on the neighbor-joining method (1000 replicates). Scale represents percentage substitution per site. Statistical support for the nodes is indicated

The *TSCD11* gene is composed of ten exons and encodes a seryl-tRNA synthetase, a 509-amino acid protein with molecular mass of approximately 56 kD. BLASTP analysis showed that TSCD11 has a seryl-tRNA synthetase (SerRs) class II catalytic domain, involving the mutated amino acid in *tscd11*. BLASTP analysis also found that TSCD11 is highly conversed in plants including *Brachypodium distachyon*, *Sorghum bicolor*, *Setaria italica*, *Triticum turgidum*, *Aegilops tauschii*, *Zea mays*, *Medicago truncatula, Cicer arietinum* and *Arachis ipaensis* (Fig. 4a). Among these species, rice TSCD11 showed the highest homology with *Sorghum bicolor* (88%). Phylogenetic analysis of TSCD11 was performed to investigate the evolutionary relationship among TSCD11 homologs. As shown in Fig. 4b, TSCD11 homologues can be divided into two clusters: monocots and dicotyledons, and OsTSCD11 belongs to monocots cluster.

### Expression of *TSCD11* Is Affected by Temperature and TSCD11 Protein Is Localized to Chloroplasts

**Fig. 5:**
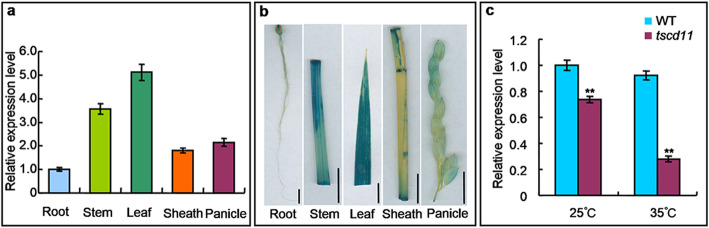
Expression pattern of *TSCD11*. **a**, qRT-PCR analysis of *TSCD11* gene in various tissues of wild-type plants. Data represent the mean ± SD (n = 3). **b**, GUS staining of root, stem, leaf, sheath and panicle in the *TSCD11* promoter-GUS transgenic plants (bars =1 cm). **c**, Relative transcript levels of *TSCD11* in the WT and *tscd11* at the 3-leaf stage grown at 25 °C and 35 °C, respectively. The rice *Histone* gene was used as an internal control. The expression level of *TSCD11* in the WT at 25 °C was set to 1.0. Data represent mean ± SD (n = 3). ** *p* < 0.01 (Student’s *t*-test)

In order to examine the expression pattern of *TSCD11* in the WT, we used qRT-PCR to investigate the transcript levels of *TSCD11* in roots, stems, leaves, sheaths and panicles. *TSCD11* was expressed in all tissues with highest expression in leaves (Fig. 5a). To further verify the tissue-specific expression of *TSCD11*, the promoter of *TSCD11* (1999 bp upstream of ATG) was amplified from wild-type genomic DNA and inserted into the binary vector pCAMBIA1305, and the construct pCAMBIA1305-*TSCD11* was then introduced into the WT by *Agrobacterium*-mediated transformation. Consisting with qRT-PCR, β-glucuronidase (GUS) staining showed the same results (Fig. 5b). To further investigate whether the mutation and temperature affect *TSCD11* expression, we detected transcript levels of *TSCD11* in 3-leaf stage seedlings of the WT and *tscd11*, and found that *TSCD11* expression was significantly lower in the *tscd11* mutant compared to the WT at 25 °C and 35 °C, with even lower in *tscd11* plants grown at 35 °C (Fig. 5c). Specifically, the expression level of *TSCD11* in the *tscd11* mutant was decreased to 73.8% and 30.4% of the WT at 25 °C and 35 °C, respectively. These results suggested that *TSCD11* expression was inhibited in the *tscd11* mutant and high temperature enhanced the inhibition.

**Fig. 6:**
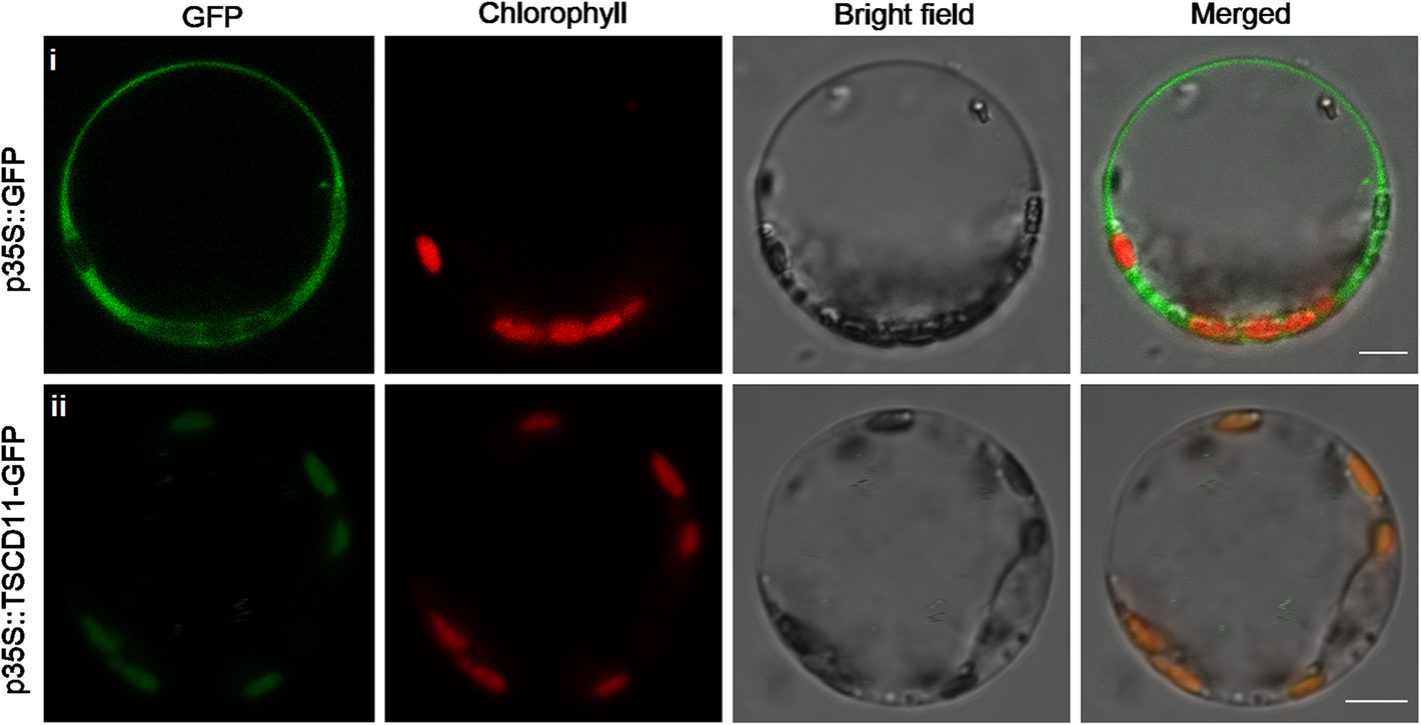
Subcellular localization of the TSCD11-GFP protein in rice protoplasts. **i**. Empty GFP as a control. **ii.** p35S::TSCD11-GFP fusion localized to chloroplast (bar = 5 μm)

The pTSCD11-GFP plasmid and the empty GFP plasmid were respectively introduced into rice protoplasts to examine subcellular localization of TSCD11. Confocal microscopy showed the TSCD11-GFP fusion protein localized to the chloroplast (Fig. 6).

### The Transcriptional Expression of Functionally Related Genes Are Affected in the *tscd11* Mutant

**Fig. 7:**
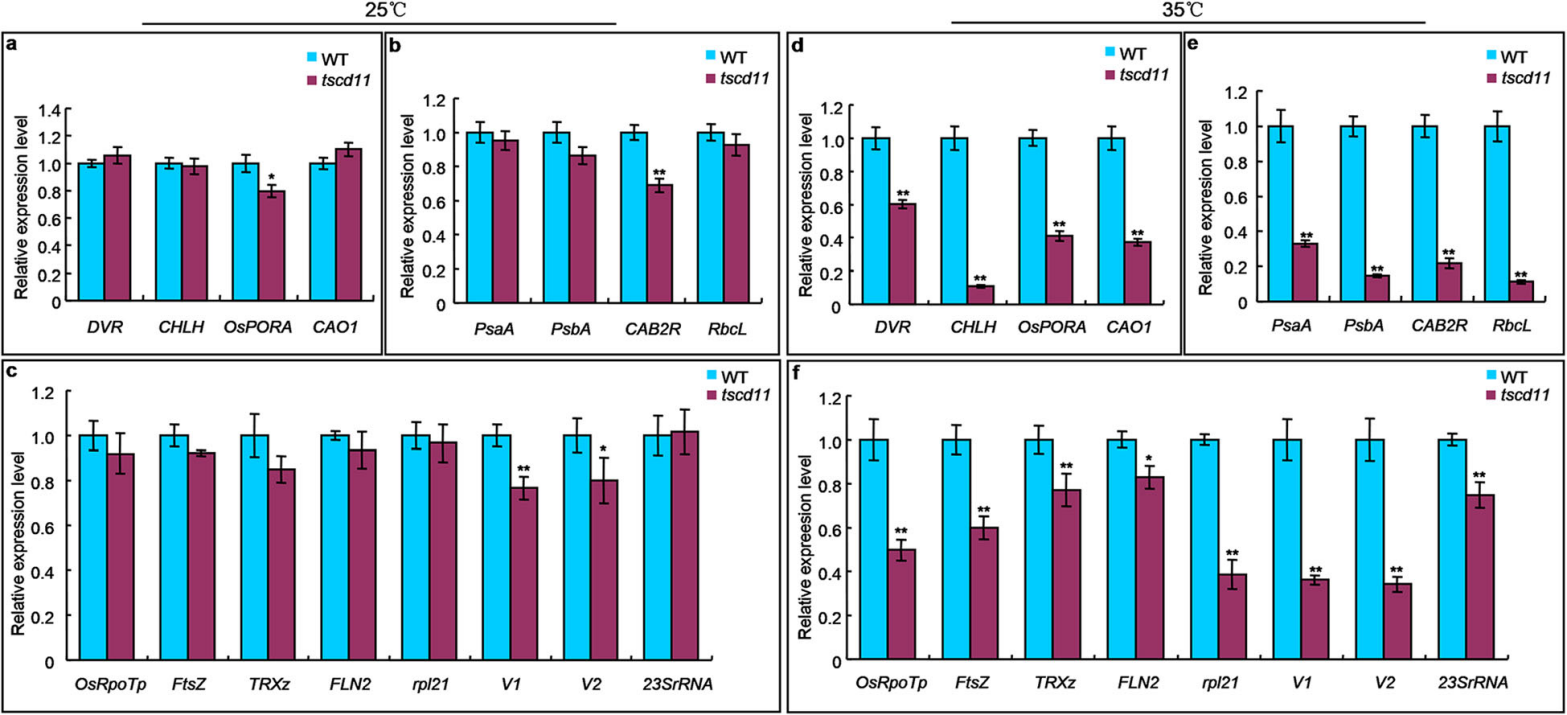
Transcriptional expression of genes related to Chl biosynthesis, photosynthesis and chloroplast development. **a**-**c** Expression of genes associated with Chl biosynthesis (**a**), photosynthesis (**b**) and chloroplast development (**c**) in WT and *tscd11* mutant at 25 °C. **d-f** Expression of genes associated with Chl biosynthesis (**d**), photosynthesis (**e**) and chloroplast development (**f**) in WT and *tscd11* mutant at 35 °C. The rice *Histone* gene was used as an internal control. The expression level of each tested gene in WT was set to 1.0. Data represent mean ± SD (n = 3). * *p* < 0.05, ** *p* < 0.01 (Student’s *t*-test)

Given that TSCD11 protein is localized to chloroplasts, the expression levels of genes related to chloroplast development might be affected. We then determined expression levels of 16 representative genes associated with Chl biosynthesis, photosynthesis and chloroplast development. At 35 °C, expression levels of Chl biosynthesis genes encoding divinyl reductase (*DVR*), Mg-chelatase H subunit (*CHLH*), protochlorophyllide oxidoreductase A (*OsPORA)* and chlorophyll a oxygenase (*CAO1*) were significantly reduced in the *tscd11* mutant compared to the WT (Fig. 7d), in accordance with the albino phenotype and decreased Chl content in *tscd11* (Fig. 1g, j). The transcript levels of photosynthesis related genes *Photosystem I Subunit A* (*PsaA*), *Photosystem II Subunit of A* (*PsbA*), *Rice Chlorophyll a/b Binding Protein (CAB2R*), *Rubisco Large Subunit* (*RbcL*) were also significantly down-regulated in *tscd11* compared with the WT (Fig. 7e). In comparison with the WT, expression levels of chloroplast development related genes *Plastid RNA Polymerase* (*OsRpoTp*), *FtsZ*, *Thioredoxinz* (*TRXz*), *PfkB-type Carbohydrate Kinase Family Protein* (*FLN2*), *Chloroplast 50S Ribosome Protein L21* (*rpl21)*, *Virescent1* (*V1*), *Virescent2* (*V2*), *23S ribosomal RNA* (*23SrRNA*) decreased significantly in *tscd11* (Fig. 7f). These results were consistent with the impaired chloroplast in the *tscd11* mutant under high temperature (Fig. 2d, h). By contrast, expression levels of almost affected genes at 35 °C in the *tscd11* mutant had little difference with those of the WT at 25 °C (Fig. 7a-c).

## Discussion

### *TSCD11* Is Essential for Chloroplast Development under High Temperature

Chloroplasts are the main organelles for photosynthesis in plants, encoding only about 100 genes (Leister 2003). Thus, the expression of these chloroplast-related genes is essential for chloroplast development. With global temperature rise overall, sudden high-temperature periods often occur in early seedling development in the field. It affects the expression of chloroplast-related genes, leading to chlorophyll deficiency/chloroplast defects, which ultimately could causes reduced yield of rice. Thus, the identification of mutants sensitive to high temperature and cloning of related gene are beneficial to elucidate the mechanism of chloroplast development at high temperature. At present, more than 80 chlorophyll deficient mutants have been identified in rice (Li et al. 2013). Six high-temperature sensitive mutants have been identified, five of which displayed an chlorotic phenotype at high temperature (over 35 °C) (Zheng et al. 2016). The *cde1(t)* mutant produced yellow green leaves at over 26 °C, while exhibited normal phenotype at lower temperatures (23 °C and 20 °C) (Liu et al. 2007). These phenotypes most likely caused by mutation of the candidate gene *OsGluRS*. The *tcm5* mutant displayed albino phenotype at 32 °C, but was normal at 20 °C (Zheng et al. 2016). The *hsa1* mutant showed albino phenotype at 32 °C, while exhibited normal phenotype at 24 °C (Qiu et al. 2017). Similar to the *tcm5* mutant, the *tscd11* mutant had albino leaves and even dead during the 4–5 leaf stage at 35 °C, while appeared normal at 25 °C. Furthermore, temperature shift experiments showed that the phenotype of *tscd11* changed significantly in both 25 °C shift to 35 °C and 35 °C shift to 25 °C, although the mutant’s phenotype could not restore completely when shifted from 35 °C to 25 °C (Additional file 1: Fig. S1f, h). Consistently, the pigment contents of *tscd11* decreased significantly compared to the WT from 35 °C to 25 °C, the pigment contents of *tscd11* were significantly higher from 35 °C to 25 °C than those at 35 °C (Additional file 1: Fig. S1g, h, k, l; Additional file 2: Table S1). Similar phenomena were also observed in *thermo-sensitive chlorophyll-deficient mutant 11* (*tcd11*) (Wang et al. 2017). These high-temperature sensitive mutants displayed different phenotypes at different temperatures, which revealed different temperature sensitive mechanisms for chloroplast development in rice. Additionally, the chloroplast of *tscd11* developed abnormally at high temperature and the transcripts of genes related to chloroplast development were also decreased in *tscd11* at 35 °C compared with WT (Fig. 7d-f). In contrast to high temperature, all transcripts of the affected genes at 35 °C in the *tscd11* at least partially restored to the WT level under low temperature (25 °C, Fig. 7a-c). These results can explain the difference in chloroplast development and leaf color between 25 °C and 35 °C. Moreover, similar to the *hsa1* mutant, the transcript levels of *TSCD11* all significantly reduced in *tscd11* compared with the WT at 25 °C and 35 °C, with even lower in *tscd11* plants grown at 35 °C. The results indicate that *TSCD11* expression is inhibited in the *tscd11* mutant and the inhibition enhanced by high temperature. In addition, the lethality of the *tscd11* mutant may be due to prevention of chloroplast development at high temperature (Fig. 2d, h). Similar to *TCM5* and *HSA1*, *TSCD11* protein is also localized to chloroplasts, confirming that the *TSCD11* gene is required for chloroplast development at high temperature.

The *TSCD11* gene encodes a seryl-tRNA synthetase, an essential enzyme for protein synthesis. Given that TSCD11 protein is localized to chloroplasts, we then determined expression levels of 9 chloroplast-encoded genes which are essential for chloroplast development. As shown in Fig. S5a, the expression levels of 7 chloroplast-encoded genes showed no significant difference between the WT and *tscd11* at 25 °C. By contrast, the expression levels of all 9 chloroplast-encoded genes decreased significantly in *tscd11* at 35 °C (Fig. S5b). And chloroplasts were observed impaired without stacked grana or stromal thylakoids in *tscd11* plants compared with the WT at 35 °C (Fig. 2d, h). These results suggested that *TSCD11* was essential in the process of chloroplast development under high temperature. According to previous studies, it was found that chloroplast development is a complex process, and the process from protoplast to mature chloroplast could be divided into three important steps. The third step involves high expression of genes related to photosynthesis (Jarvis and López-Juez 2013). *PsaA*, *PsbA*, *CAB2R* and *RbcL* are typical genes involved in the third step. Because their expression were all declined significantly at 35 °C in *tscd11* compared to the WT (Fig. 7e), the mutation in *TSCD11* may affect the expression of photosynthesis related genes under high temperature.

### ROS Induces the Expression of Genes Related to ROS Detoxification

As the Chloroplasts impaired, the plants are often weakness in growth. And the weakness plants are sensitive to ROS, especially under stress situation (Chen et al. 2018). The common stress situations include high temperature or high light. ROS acted as a signal to induce change in genes expression (Baxter et al. 2014; Triantaphylidès 2008). To verify it in the *tscd11* mutant, we measured contents of ROS-related substances. At 25 °C, contents of H_2_O_2_ and MDA, and CAT activity had no difference between the WT and the *tscd11* mutant (Additional file 1: Fig. S3c-e). However, at 35 °C, contents of H_2_O_2_ and MDA were notably increased in *tscd11*, and the activity of CAT was significantly decreased compared to the WT (Additional file 1: Fig. S3h-j). Interestingly, the expression of genes related to ROS detoxification, such as *AOX1a*, *AOX1b*, *APX1*, *CATB*, *SODA1* and *SODB* were significantly induced in the *tscd11* mutant. Although the expression levels of all antioxidant genes were dramatically increased at 35 °C (Additional file 1: Fig. S3l), excess ROS still accumulated in *tscd11* at high temperature (Additional file 1:Fig. S3h-j). Similar phenomena were also observed in the *ABA overlysensitive5* (*abo5*), *local lesions 1* (*ls1*), and *slow growth 1* (*slg1*) mutants (Liu et al. 2010; Qiu et al. 2019; Yuan and Liu 2012). Therefore, it is possible that increased expression of those genes may not be sufficient for reducing ROS to normal levels in the *tscd11* mutant at high temperature.

## Conclusion

The *TSCD11* encodes a chloroplast-targeted seryl-tRNA synthetase which is essential for early chloroplast development, especially at high temperature. These results provide basis for further study on the molecular mechanism of chloroplast development under high temperature.

## Methods

### Plant Materials and Growth Conditions

In this study, *tscd11* (*temperature -sensitive chlorophyll*-*deficient mutant 11*) was obtained from *japonica* variety Wuyujing7 (WT) by ethyl methane sulfonate (EMS). An F_2_ segregating population was derived from a cross between the *tscd11* and *indica* cultivar 93–11 for fine mapping *TSCD11* locus. All rice plants were planted in the paddy field during the normal season in two different successive months (May and June) at Hangzhou. In the F_2_ population, the plants with albino phenotype were selected for genetic mapping. For temperature treatment experiments, the WT and *tscd11* seeds were grown in growth chambers (12 h of light and 12 h of dark; light intensity 200 μmol m^− 2^ s^− 1^) at different temperatures (25 °C, 30 °C and 35 °C). For temperature transition experiments of 25 °C shift to 35 °C and 35 °C shift to 25 °C, the WT and *tscd11* seeds were grown in growth chambers (12 h of light and 12 h of dark; light intensity 200 μmol m^− 2^ s^− 1^) at the temperature of 25 °C or 35 °C till the 2-leaf stage, and then grown in growth chambers (12 h of light and 12 h of dark; light intensity 200 μmol m^− 2^ s^− 1^) at the temperature of 35 °C or 25 °C from the 2-leaf stage to the 3-leaf stage. The wild-type and *tscd11* seeds were grown hydroponically in nutrient solution in growth chambers as previously described (Ueno et al. 2009).

### Pigment Content and Agronomic Traits Measurent

Fresh leaves (0.05 g) were sampled from the WT and *tscd11* plants at the 3-leaf stage grown in growth chambers and in the paddy field in different growth periods, and cut into small pieces, respectively. These leaves were completely immersed in 5 ml 95% ethanol with three biological repeats and keep them in dark at 26 °C until 24 h later. The contents of chlorophyll *a* (Chl *a*), chlorophyll *b* (Chl *b*), and carotenoids (Car) were determined according to the methods described by Arnon (1949) and Wellburn (1994) Taking 1 ml samples solution were measured at 663 nm, 645 nm and 470 nm, using ultraviolet spectrophotometer (DU800, Beckman, USA). Wild-type and *tscd11* mutant plants were grown in experimental rice fields of Hangzhou. And we surveyed some agronomic traits of the wild-type and *tscd11* plants at maturity (Additional file 2: Table S2).

### Transmission Electron Microscopy Analysis

Fresh leaves of the wild-type and *tscd11* plants at the 3-leaf stage grown at 25 °C or 35 °C in growth chambers were collected and cut into small pieces. Then samples were fixed in 2.5% glutaraldehyde in phosphate buffer (pH = 7.4) at 4 °C for 4 h after vacuum and then in 1% (w/v) OsO_4_. TEM assays were done according to the method described by Gothandam et al. (2005). The samples were examined with a Hitachi H-7650 transmission electron microscope.

### Histochemical Staining, Determination of ROS-Related Physiological Index and TUNEL Assay

Superoxide anion was detected by NBT, DAB staining according to the method described by Thordal-Christensen et al. (2010). H_2_O_2_, MDA contents and CAT activity of WT and *tscd11* were measured as described by Moradi and Ismail (2007). The TUNEL assay was performed with Fluorescein in Situ Cell Death Detection Kit (Roche), according to the method of Huang et al. (2007). The leaves of WT and *tscd11* were fixed with FAA fixative and embedded in paraffin. The sections were microscopically examined to select slides and dewaxed by gradient alcohol. The slides were immersed in 4% methanol-free formaldehyde in PBS, and tissue sections were permeabilized with proteinase K. Equilibration Buffer was then added and covered with plastic coverslip. After incubation in another buffer containing Equlibration Buffer, Nucleotide Mix and rTdT Enzyme at 37 °C in the dark for 1 h, the slides were dipped in 2 × SSC without plastic coverslip. Cells were then stained with PI and the localized green fluorescence (520 nm) of apoptotic cells (fluorescein-12-dUTP) in a red fluorescence (620 nm) background (PI) were detected by confocal fluorescence scanning microscope (LSM700, Carl Zeiss, Inc., USA). Histochemical staining, determination of ROS-related physiological index and TUNEL assay were performed on the top leaves from the wild-type and *tscd11* plants at the 3-leaf stage, which were grown at 25 °C or 35 °C in growth chambers.

### Genetic Analysis and Map-Based Cloning of *TSCD11*

For the genetic analysis, crosses were performed between the *tscd11* mutant and Wuyujing7 (*Japonica* variety) or 93–11 cultivar (*indica* variety), respectively. For fine mapping of *TSCD11* locus, 197 mutant individuals were selected from F_2_ population to map the *TSCD11* locus. The genomic DNA was extracted from F_2_ plants, according the CTAB method (Murray and Thompson 1980) SSR markers were obtained from the Gramene database (http://www.gramene.org). In order to narrow the target interval, new polymorphic markers were designed, which were based on the sequence differences between the *japonica* rice cultivar Nipponbare and the *indica* cultivar 93–11.These markers are listed in Additional file 2: Table S4.

### Vector Construction and Transformation

For complementation of the *tscd11* mutant, a 6449-bp genomic DNA fragment containing entire *TSCD11* coding region, 1999-bp upstream sequence and 846-bp downstream sequence was amplified by PCR using the primers TSCD11-COM F/R. PCR products were digested with Kpn I and BamH I. The target fragment was purified and cloned into the binary vector pCAMBIA1300. The pCAMBIA1300-TSCD11 vector and control vector (pCAMBIA1300, CK) were introduced into the *tscd11* mutant by Agrobacterium-mediated. The 1999-bp sequence of *TSCD11* promoter was amplified by PCR using the primers TSCD11-GUS F/R. PCR products were digested with Hind III and Nco I. The fragment was cloned into the binary vector pCAMBIA1305. Primers used for vector construction are listed in Additional file 2: Table S5.

### Phylogenetic Analysis

The full-length sequence of TSCD11 protein was obtained using Gramene (http://www.gramene.org). The blast analysis was performed to find homologues of TSCD11 on the NCBI website (NCBI, http//www.ncbi.nlm.nih.gov). Multiple sequences were aligned and a phylogenetic tree was constructed by MEGA version 6.0 software based on the neighbor-joining method (1000 replicates).

### Subcellular Location of TSCD11 in Rice Protoplast

To investigate the subcellular localization of TSCD11, the *TSCD11* full-length cDNA without termination codon was cloned into the GFP vector pCA1301-35S-S65T-GFP (Ren et al. 2017). We amplified the full-length *TSCD11* coding sequence without the termination codon with primers TSCD11-GFP F/R (Additional file 2: Table S5). According the previous protocols, the construct (p35S:: TSCD11-GFP) and control vector were transformed into rice chloroplast (Yu et al. 2014). Fluorescence signals were observed with confocal fluorescence scanning microscope (LSM700, Carl Zeiss, Inc., USA).

### RNA Extraction and Quantitative Real Time PCR (qRT-PCR) Analysis

To understand the expression pattern of *TSCD11*, Total RNA was extracted from roots, stems, leaves, sheaths and panicles of wild-type plants grown in the paddy field. For transcriptional expression analysis of *TSCD11* and 36 genes (*WRKY72*, *Osh36*, *SGR*, *WRKY24*, *WRKY70*, *AOX1a*, *AOX1b*, *APX1*, *CATB*, *SODA1*, *SODB*, *DVR*, *CHLH*, *OsPORA*, *CAO1*, *psaA*, *psbA*, *CAB2R*, *RbcL*, *OsRpoTp*, *FtsZ*, *TRXz*, *FLN2*, *rpl21*, *V1*, *V2*, *23SrRNA*, *AtpB*, *AtpE*, *16SrRNA*, *rpl23*, *rps7*, *rps10*, *rps18*, *rps19*, *rps20*) associated with senescence, ROS-responsive, detoxification, Chl biosynthesis, photosynthesis and chloroplast development, total RNA was obtained from the fresh leaves of the WT and *tscd11* plants at the 3-leaf stage grown at 25 °C or 35 °C in growth chambers.

Total RNA was extracted using the AxyPrep total RNA Miniprep Kit (Axygen) according to the manufacturer’s instructions. The extracted RNA was reverse-transcribed using the Rever TraAce quantitative PCR RT Master Mix Kit with gDNA remover (Toyobo) according to the manufacturer’s instructions. qRT-PCR experiment was performed on the ABI PRISM 7900HT Sequence Detector (Applied Biosystems). All primers sequences for qRT-PCR are listed in Additional file 2: Table S6. The rice *Histone* gene was used as an internal control. The expressed data were the means of three biological replicates. A Student’s *t*-test was performed for statistical analysis.

## Supplementary information

**Additional file 1 Fig. S1** Comparison of phenotypes and pigment contents between the wild-type and *tscd11* plants. **a-d** Phenotypes of the WT and *tscd11* at the 2-leaf stage grown at continuous 25 °C (**a, b**) and 35 °C (**c, d**), respectively. **e-h** Phenotypes of the WT and *tscd11* at the 3-leaf stage grown at continuous 25 °C, shift 25 °C to 35 °C, continuous 35 °C, shift 35 °C to 25 °C, respectively. Bar = 2 cm. **i-l** Pigment contents of the new third leaf of the wild-type and *tscd11* plants grown at continuous 25 °C, shift 25 °C to 35 °C, continuous 35 °C, shift 35 °C to 25 °C at the 3-leaf stage. Data represent mean ± SD (*n* = 3). * *p* < 0.05, ** *p* < 0.01 (Student’s *t*-test). **Fig. S2** Phenotypes and pigment contents comparison between wild-type and *tscd11* plants. **a**, **d** Phenotypes of the WT and *tscd11* at the tillering (bar = 12 cm), and heading stage (bar = 10 cm), respectively, in the paddy field. **b**, **e** Close-up image of wild-type leaf and *tscd11* leaf at the tillering, and heading stage (bar = 5 cm), respectively. **c**, **f** Pigment contents of WT and *tscd11* at the tillering, and heading stage, respectively. Data represent mean ± SD (n = 3). * *p* < 0.05, ** *p* < 0.01 (Student’s *t*-test). **Fig. S3** ROS accumulation and genomic DNA fragmentation in the *tscd11* mutant. **a**, **b**, **f, g** DAB (**a, f**) and NBT (**b, g**) staining of leaves from wild-type and *tscd11* plants at 25 °C (**a, b**) and 35 °C (**f, g**), respectively (bar =1 cm). **c**-**e**, **h**-**j** Statistic analysis of H_2_O_2_ content (**c**, **h**), MDA content (**d**, **i**), CAT activity (**e**, **j**) at 25 °C (**c-e**) and 35 °C (**h-j**), respectively. **k**, **l** Relative expression levels of senescence, ROS-responsive and ROS detoxification related genes in wild-type and *tscd11* plants at 25 °C (**k**) and 35 °C (**l**) The rice *Histone* gene was used as an internal control. The expression level of each tested genes in WT was set to 1.0. Data represent mean ± SD (n = 3). * *p* < 0.05, ** *p* < 0.01 (Student’s *t*-test). **Fig. S4** TUNEL assay of wild-type and *tscd11* leaves at 25 °C and 35 °C. Red signal is PI staining, green color represents positive result of apoptotic cells (bar = 50 μm). **a**, **b** leaves from wild-type and *tscd11* plants at 25 °C. **c**, **d** leaves from wild-type and *tscd11* plants at 35 °C. **Fig. S5** Transcriptional expression of chloroplast-encoded genes. **a,** Expression of chloroplast-encoded genes in the WT and *tscd11* mutant at 25 °C. **b,** Expression of chloroplast-encoded genes in the WT and *tscd11* mutant at 35 °C. The rice *Histone* gene was used as an internal control. The expression level of each tested gene in the WT was set to 1.0. Data represent mean ± SD (n = 3). * *p* < 0.05, ** *p* < 0.01 (Student’s *t*-test).

**Additional file 2 Table S1** Pigment contents of the WT and the *tscd11* mutant **Table S2** Agronomic characters of the WT and the *tscd11* mutant. **Table S3** Genetic analysis of *tscd11*. **Table S4** Primers used for fine mapping. **Table S5** Primers used for vector construction. **Table S6** Primers used for qRT-PCR.

## Data Availability

All relevant data are provided as figures or tables within the paper.

## Abbreviations

AARSs: Aminoacyl-tRNA synthetases
*AOX1a, AOX1b*: *Alternative Oxidase*
*APX1*: *Ascorbate Peroxidase*
*AtpB*: *ATP synthase F1 beta subunit 2*
*CAB2R*: *Rice Chlorophyll a/b Binding Protein*
*CAO1*: *Chlorophyll a Oxygenase*
Car: Carotenoid
CAT: Catalase
*CATB*: *Catalase*
Chl: Chlorophyll
Chl *a*: Chlorophyll *a*
Chl *b*: Chlorophyll *b*
*CHLH*: *Mg-chelatase H subunit*
DAB: 3, 3′-diaminobenzidine
*DVR*: *Divinyl Reductase*
*FLN2*: *PfkB-type Carbohydrate Kinase Family Protein*
H_2_O_2_: Hydrogen peroxide
MDA: Malondialdehyde
NBT: Nitro blue tetrazolium
O_2_^−^: superoxide anions
*OsPORA*: *Protochlorophyllide Oxidoreductase A*
*OsRpoTp*: *Plastid RNA Polymerase*
PI: Propidium iodide
*PsaA*: *Photosystem I Subunit A*
*PsbA*: *Photosystem II Subunit of A*
qRT-PCR: quantitative real time PCR
*RbcL*: *Rubisco Large Subunit*
ROS: Reactive oxygen species
*rpl21*: *Chloroplast 50S Ribosome Protein L21*
*rpl23*: *Chloroplast 50S Ribosome Protein L23*
*SGR*: *Stay Green Rice*
*SODA1, SODB*: *Superoxide Dismutase*
*23SrRNA*: *23S ribosomal RNA*
TEM: Transmission electron microscopy
*TRXz*: *Thioredoxinz*
TUNEL: terminal deoxynucleotidyl transferase-mediated dUTP nick-end labeling
*V1*: *Virescent1*
*V2*: *Virescent2*
*WRKY72, WRKY24, WRKY70*: *WRKY transcription factor*
WT: Wild type
