# Supplementary material for: Isolation of TSCD11 Gene for Early Chloroplast Development under High Temperature in Rice
Source: Rice (N Y). 2020 Jul 17;13:49. doi: 10.1186/s12284-020-00411-6 (PMC7367945; doi:10.1186/s12284-020-00411-6)
Supplement: Supplementary file 1 — Additional file 1 Fig. S1 Comparison of phenotypes and pigment contents between the wild-type and tscd11 plants. a-d Phenotypes of the WT and tscd11 at the 2-leaf stage grown at continuous 25 °C (a, b) and 35 °C (c, d), respectively. e-h Phenotypes of the WT and tscd11 at the 3-leaf stage grown at continuous 25 °C, shift 25 °C to 35 °C, continuous 35 °C, shift 35 °C to 25 °C, respectively. Bar = 2 cm. i-l Pigment contents of the new third leaf of the wild-type and tscd11 plants grown at continuous 25 °C, shift 25 °C to 35 °C, continuous 35 °C, shift 35 °C to 25 °C at the 3-leaf stage. Data represent mean ± SD (n = 3). * p < 0.05, ** p < 0.01 (Student’s t-test). Fig. S2 Phenotypes and pigment contents comparison between wild-type and tscd11 plants. a, d Phenotypes of the WT and tscd11 at the tillering (bar = 12 cm), and heading stage (bar = 10 cm), respectively, in the paddy field. b, e Close-up image of wild-type leaf and tscd11 leaf at the tillering, and heading stage (bar = 5 cm), respectively. c, f Pigment contents of WT and tscd11 at the tillering, and heading stage, respectively. Data represent mean ± SD (n = 3). * p < 0.05, ** p < 0.01 (Student’s t-test). Fig. S3 ROS accumulation and genomic DNA fragmentation in the tscd11 mutant. a, b, f, g DAB (a, f) and NBT (b, g) staining of leaves from wild-type and tscd11 plants at 25 °C (a, b) and 35 °C (f, g), respectively (bar =1 cm). c-e, h-j Statistic analysis of H2O2 content (c, h), MDA content (d, i), CAT activity (e, j) at 25 °C (c-e) and 35 °C (h-j), respectively. k, l Relative expression levels of senescence, ROS-responsive and ROS detoxification related genes in wild-type and tscd11 plants at 25 °C (k) and 35 °C (l) The rice Histone gene was used as an internal control. The expression level of each tested genes in WT was set to 1.0. Data represent mean ± SD (n = 3). * p < 0.05, ** p < 0.01 (Student’s t-test). Fig. S4 TUNEL assay of wild-type and tscd11 leaves at 25 °C and 35 °C. Red signal is PI staining, gree [file 12284_2020_411_MOESM1_ESM.doc]

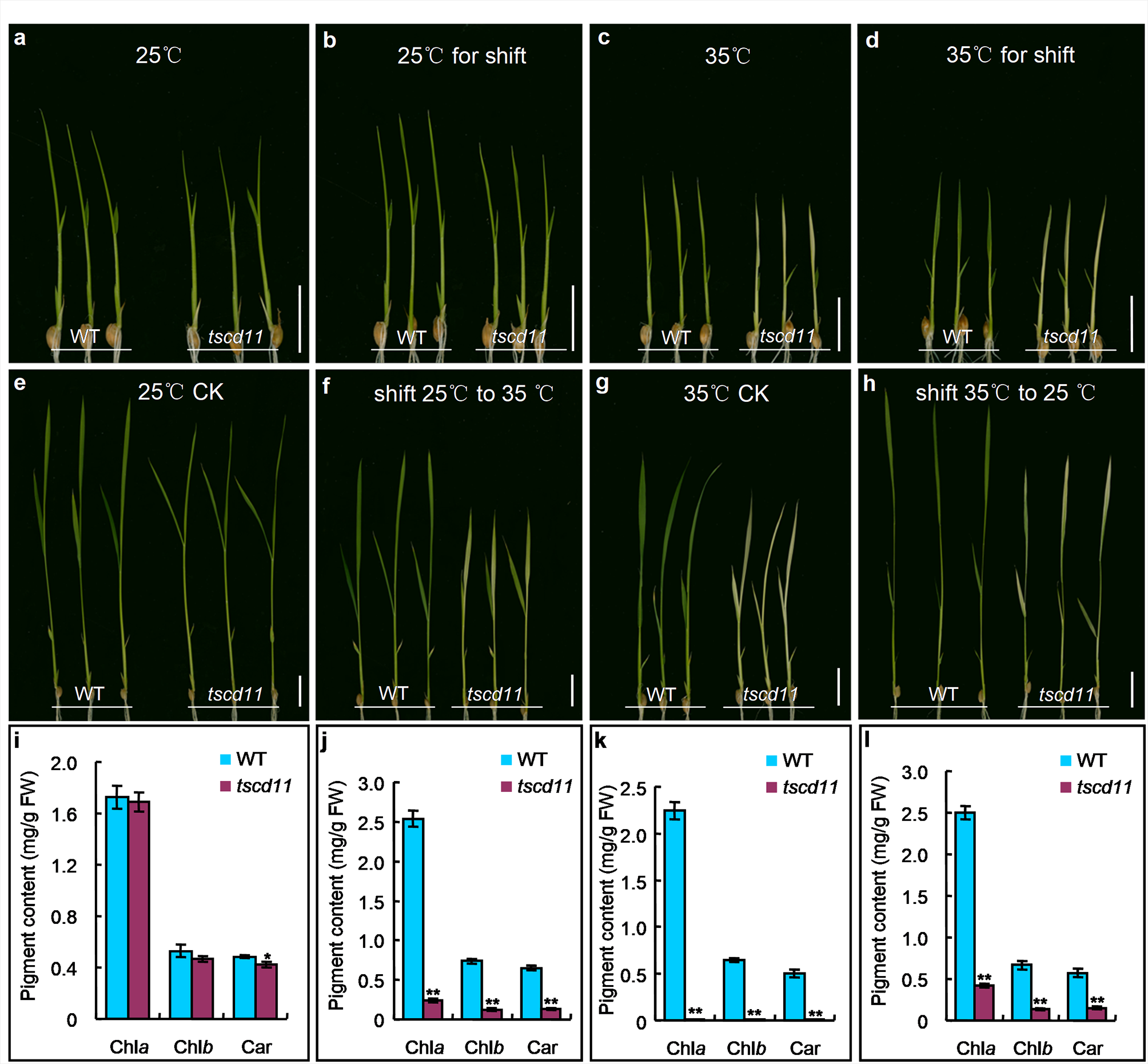


**Fig. S1** Comparison of phenotypes and pigment contents between the wild-type and *tscd11* plants. **a-d** Phenotypes of the WT and *tscd11* at the 2-leaf stage grown at continuous 25°C (**a, b**) and 35°C (**c, d**), respectively. **e-h** Phenotypes of the WT and *tscd11* at the 3-leaf stage grown at continuous 25°C, shift 25°C to 35°C, continuous 35°C, shift 35°C to 25°C, respectively. Bar = 2 cm. **i-l** Pigment contents of the new third leaf of the wild-type and *tscd11* plants grown at continuous 25°C, shift 25°C to 35°C, continuous 35°C, shift 35°C to 25°C at the 3-leaf stage. Data represent mean ± SD (n = 3). * *p*<0.05, ** *p*<0.01 (Student’s *t*-test).


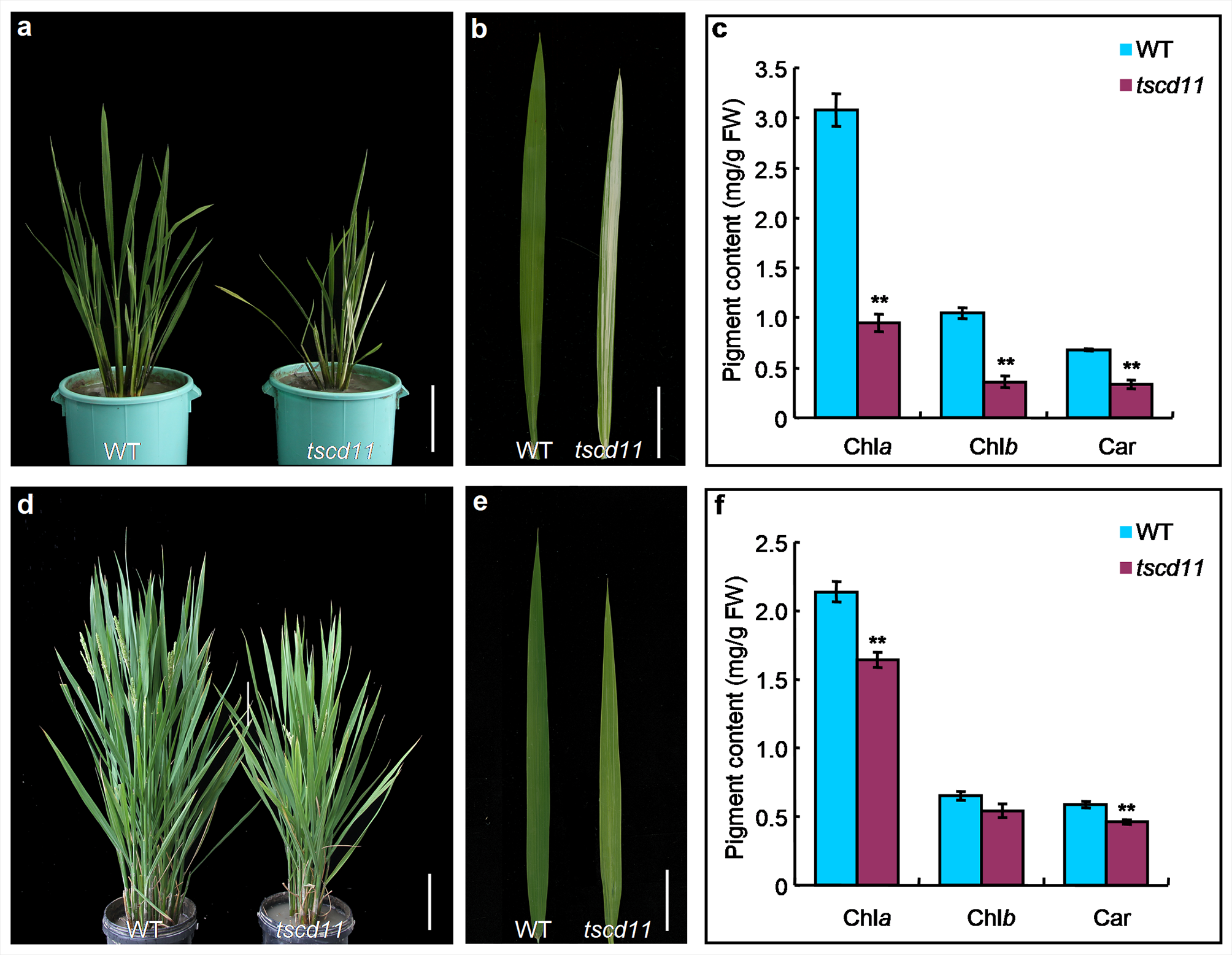


**Fig. S2** Phenotypes and pigment contents comparison between wild-type and *tscd11* plants. **a**, **d** Phenotypes of the WT and *tscd11* at the tillering (bar = 12 cm), and heading stage (bar = 10cm), respectively, in the paddy field. **b**, **e** Close-up image of wild-type leaf and *tscd11* leaf at the tillering, and heading stage (bar = 5 cm), respectively. **c**, **f** Pigment contents of WT and *tscd11* at the tillering, and heading stage, respectively. Data represent mean ± SD (n = 3). * *p*<0.05, ** *p*<0.01 (Student’s *t*-test).

**
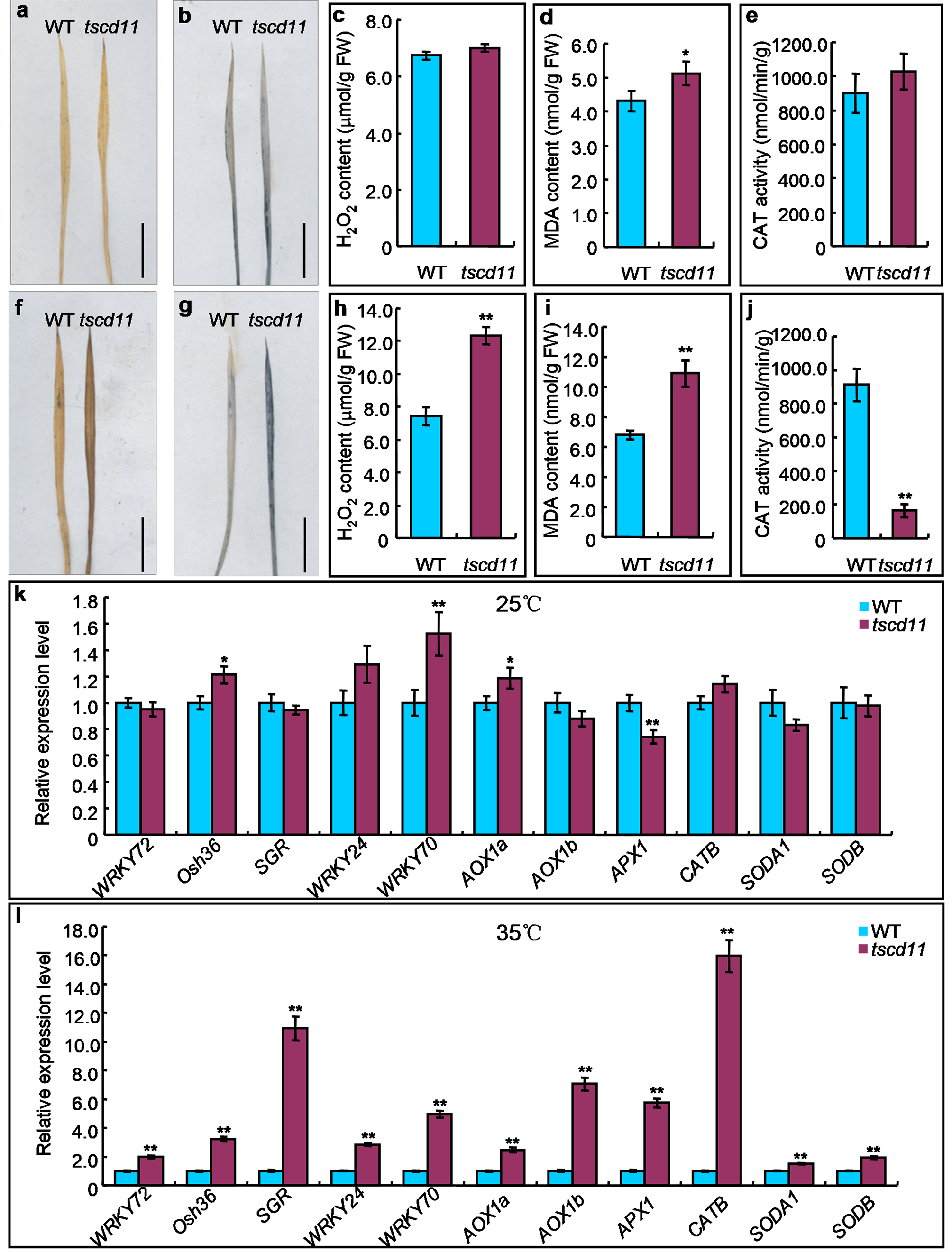
**

**Fig.** **S3** ROS accumulation and genomic DNA fragmentation in the *tscd11* mutant. **a**, **b**, **f, g** DAB (**a, f**) and NBT (**b, g**) staining of leaves from wild-type and *tscd11* plants at 25°C (**a, b**) and 35°C (**f, g**), respectively (bar =1 cm). **c**-**e**, **h**-**j** Statistic analysis of H_2_O_2_ content (**c**, **h**), MDA content (**d**, **i**), CAT activity (**e**, **j**) at 25°C (**c-e**) and 35°C (**h-j**), respectively. **k**, **l** Relative expression levels of senescence, ROS-responsive and ROS detoxiﬁcation related genes in wild-type and *tscd11* plants at 25°C (**k**) and 35°C (**l**) The rice *Histone* gene was used as an internal control. The expression level of each tested genes in WT was set to 1.0. Data represent mean ± SD (n = 3). * *p*<0.05, ** *p*<0.01 (Student’s *t*-test).


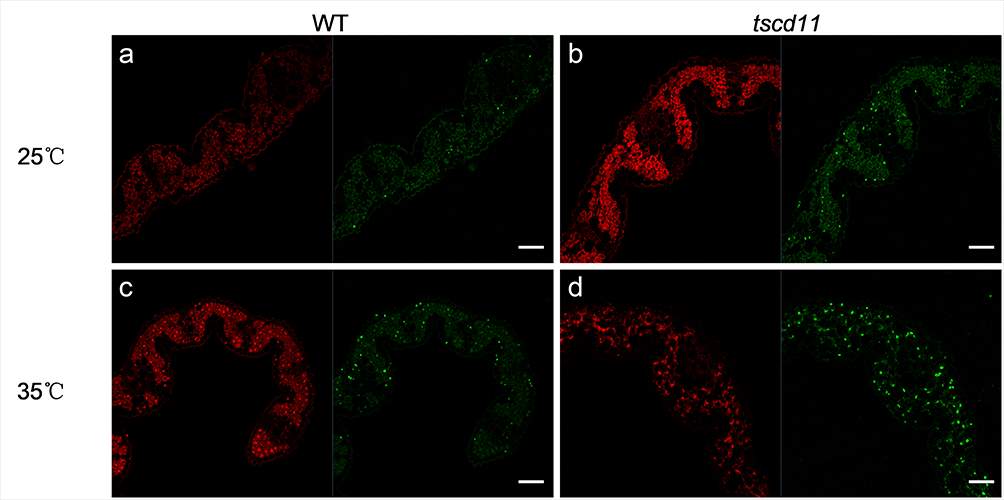


**Fig.** **S4** TUNEL assay of wild-type and *tscd11* leaves at 25°C and 35°C. Red signal is PI staining, green color represents positive result of apoptotic cells (bar = 50 μm). **a**, **b** leaves from wild-type and *tscd11* plants at 25°C. **c**, **d** leaves from wild-type and *tscd11* plants at 35°C.


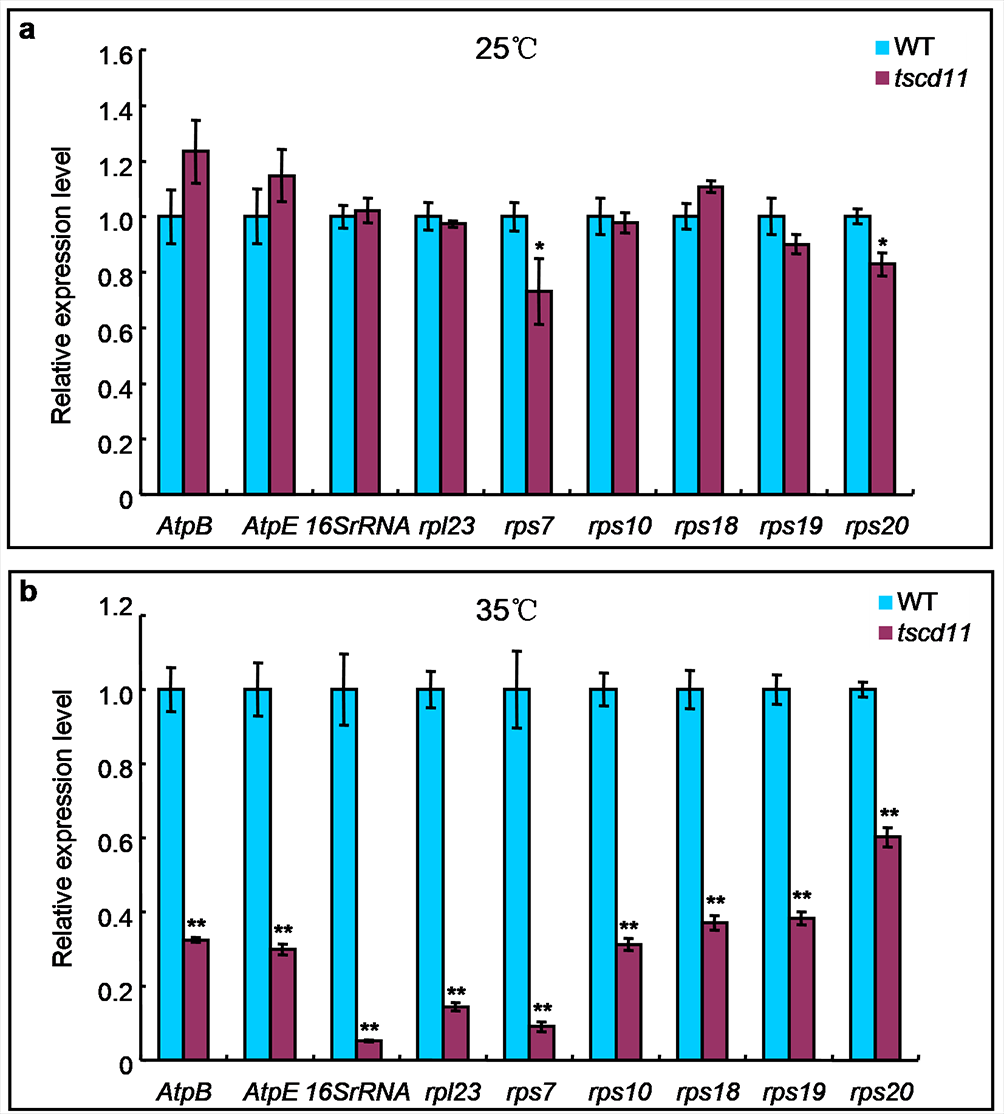


**Fig.** **S5** Transcriptional expression of chloroplast-encoded genes. **a,** Expression of chloroplast-encoded genes in the WT and *tscd11* mutant at 25°C. **b,** Expression of chloroplast-encoded genes in the WT and *tscd11* mutant at 35°C. The rice *Histone* gene was used as an internal control. The expression level of each tested genes in the WT was set to 1.0. Data represent mean ± SD (n = 3). * *p*<0.05, ** *p*<0.01 (Student’s *t*-test).
