# Supplementary material for: Isolation of TSCD11 Gene for Early Chloroplast Development under High Temperature in Rice
Source: Rice (N Y). 2020 Jul 17;13:49. doi: 10.1186/s12284-020-00411-6 (PMC7367945; doi:10.1186/s12284-020-00411-6)
Supplement: Supplementary file 2 — Additional file 2 Table S1 Pigment contents of the WT and the tscd11 mutant Table S2 Agronomic characters of the WT and the tscd11 mutant. Table S3 Genetic analysis of tscd11. Table S4 Primers used for fine mapping. Table S5 Primers used for vector construction. Table S6 Primers used for qRT-PCR. [file 12284_2020_411_MOESM2_ESM.doc]

**Table S1. Pigment contents of the WT and the *tscd11*** mutant

|  | WT | |  | *tscd11* | |
| --- | --- | --- | --- | --- | --- |
|  | 35°C | from 35°C to 25°C |  | 35°C | from 35°C to 25°C |
| Chl *a* | 2.242 ± 0.092 | 2.501 ± 0.083* |  | 0.015 ± 0.002 | 0.424 ± 0.021** |
| Chl *b* | 0.646 ± 0.022 | 0.669 ± 0.050 |  | 0.009 ± 0.001 | 0.134 ± 0.010** |
| Car | 0.502 ± 0.039 | 0.573 ± 0.056 |  | 0.014 ± 0.002 | 0.152 ± 0.014** |

Data represent the mean ± SD (n = 3). * *p*<0.05, ** *p*<0.01 (Student’s *t*-test).

**Table S2. Agronomic traits of the WT and the *tscd11* mutant**

| Agronomic traits | WT | *tscd11* |
| --- | --- | --- |
| Plant height/cm | 96.94 ± 1.59 | 76.51 ± 2.32** |
| Tiller number | 7.89 ± 0.93 | 9.11 ± 0.78** |
| Length of flag leaf/cm | 27.86 ± 3.17 | 22.00 ± 1.09** |
| Length of the second leaf from up to down/cm | 32.33 ± 2.49 | 32.52 ± 1.17 |
| length of the third leaf from up to down/cm | 35.87 ± 2.24 | 31.68 ± 0.62** |
| Width of flag leaf/cm | 1.71 ± 0.09 | 1.54 ± 0.11** |
| Width of the second leaf from up to down/cm | 1.47 ± 0.08 | 1.37 ± 0.04** |
| Width of the third leaf from up to down/cm | 1.43 ± 0.05 | 1.29 ± 0.06** |
| Panicle length/cm | 16.71 ± 0.49 | 15.55 ± 0.56** |
| No. of primary rachis branches | 15.89 ± 0.78 | 15.50 ± 0.83 |
| No. of secondary rachis branches | 22.12 ± 1.66 | 18.50 ± 1.26** |
| Grain length/cm | 7.47 ± 0.28 | 7.26 ± 0.38 |
| Grain width/cm | 3.51 ± 0.27 | 3.35 ± 0.26* |
| 1000-grain weight/g | 28.30 ± 0.09 | 25.16 ± 0.08** |
| Seed-setting rate/% | 94.85 ± 1.56 | 81.04 ± 4.64** |

Data represent the mean ± SD (n = 9). * *p*<0.05, ** *p*<0.01 (Student’s *t*-test).

**Table S3. Genetic analysis of *tscd11***

| Cross | F_1_ | F_2_ | | *χ^2^* (3:1) | *P* value |
| --- | --- | --- | --- | --- | --- |
|  |  | Wild-type | Mutant |  |  |
| *tscd11*/Wuyujing7 | Normal | 276 | 85 | 0.4072 | 0.5233 |
| *tscd11*/93-11 | Normal | 365 | 107 | 1.3672 | 0.2423 |

**Table S4. Primers used** for fine mapping

| Marker | Forward primer sequence (5’-3’) | Reverse primer sequence (5’-3’) |
| --- | --- | --- |
| B11-12 | TGAACCCTGCTCTTCTGAGTC | AAAGAAGATATGAAGGCACCG |
| B11-13 | CTTCATAAATGATACATGGTGTC | TTTTGGGATTGTCCTTCTTCG |
| P1 | TTGGCAAACTCCAAGTGTGA | AAAAGGGGTAGAGGGAGGGT |
| P2 | AAGAACCACTCAAACCCCAC | ATGAGCAGAATGTTTGGATGT |
| P3 | AACGCGCAAAAGTTCAATTT | TCTAACGACCCACATTGTTT |
| P4 | TAATGTTCTGGCTGTCAGGG | GCCGCCACCTACATTATTCA |
| P5 | ACGTACCAACATGAATCAGGA | TCTTGATTAATCCCAAGACTGGA |

**Table S5. Primers used for vector construction**

| Primer | Sequence（5’- 3’） | Restriction enzyme |
| --- | --- | --- |
| TSCD11-COM-F | acgaattcgagctcggtacccgatttcctccataactgtgca | Kpn Ⅰ |
| TSCD11-COM-R | tcgactctagaggatcccccaatcaatcgctagcaaaac | BamH Ⅰ |
| TSCD11-GUS-F | GCAGGCATGCAAGCTTcgatttcctccataactgtgca | Hind Ⅲ |
| TSCD11-GUS-R | CTCAGATCTACCATGGctcgccggagaggaggcggcgg | Nco Ⅰ |
| TSCD11-GFP-F | TTACAATTACAGTCGAATGCTGACCTGCGGCCGCTTCC | Sal Ⅰ |
| TSCD11-GFP-R | TGGATCCTCTAGAGTCTTTGGTTTTCGGGGAGAGCACT |  |

**Table S6. Primers used for qRT-PCR**

| Gene | Forward primer sequence (5’-3’) | Reverse primer sequence (5’-3’) |
| --- | --- | --- |
| *WRKY72* | CACCACAAATCACATCTACTCCG | GCTGAAGGGAAGAGAGGTGAG |
| *Osh36* | GCACGGAGGCGAACGA | TTGAGCGGTAGCACCCATT |
| *SGR* | AGGGGTGGTACAACAAGCTG | GCTCCTTGCGGAAGATGTAG |
| *WRKY24* | GTACATGAGCCAGCACCAG | TCAGTAGAGCGAGTTCTGGA |
| *WRKY70* | GACGGGAGCGTCTTACTCTT | GGCTGCTCAAAGAACGACAT |
| *AOX1a* | CTTCGCATCGGACATCCATTA | TCCTCGGCAGTAGACAAACATC |
| *AOX1b* | CCTGCTCAGTTCATCACCATCA | GCATAAAACGGAGTGACAATAGC |
| *APX1* | AGGTGCCACAAGGAAAGATCTGGT | TCAGCAGGGCTTTGTCACTAGGAA |
| *CATB* | GCTTGCTTTCTGCCCAGCGATAAT | AAATAGTTTGGGCCAAGACGGTGC |
| *SODA1* | ATCTGGATGGGTGTGGCTAGCTTT | AGTACGCATGCTCCCAGACATCAA |
| *SODB* | TCCGCCGTATAAACTTGATGCCCT | TGGGTTGCCGTTGTTGTATGCTTC |
| *TSCD11* | AGTGGCTCGAAGTTCTACTACT | ACCTGAGTATTTTGGGCCCTTG |
| *DVR* | CGAGCCCAGGTTCATCAAGGTGC | CCTCCCGATCTTGCCGAACTCC |
| *CHLH* | AACTGGATGAGCCAGAAGAGA | AAATGCAAAAGACTTGCGACT |
| *OsPORA* | ATGGCTCTCCAAGTTCAG | TGGCTCACGCTAAGGAAC |
| *CAO1* | GACACCTTCATCTGGGCTTCAA | CGAGAGACATCCGGTAGAGC |
| *psaA* | GCGAGCAAATAAAACACCTTTC | GTACCAGCTTAACGTGGGGAG |
| *psbA* | CCCTCATTAGCAGATTCGTTTT | ATGATTGTATTCCAGGCAGAGC |
| *CAB2R* | TGTTCTCCATGTTCGGCTTCT | GCTACGGTCCCCACTTCACT |
| *RbcL* | CTTGGCAGCATTCCGAGTAA | ACAACGGGCTCGATGTGATA |
| *OsRpoTp* | AAGCAGACAGTGATGACATC | ATCACATGCATGCACCCAAA |
| *FtsZ* | AAAGGACATAACCTTGCAAG | AGTTTTCCTATTGAACCGTG |
| *TRXz* | TGTGAAGGTGGATACTGATGATGA | GTCTTTGCTTTGATCTGGACTGAA |
| *FLN2* | AGGAGCCATTTACATTATAAGCC | AACTTACGTTTCGGTTGAGCA |
| *rpl21* | AAGAAGAGGAGGCTGCGGT | GACATTGGCGCCTTTCAGC |
| *V1* | AGAATCAGCGCGAGAAGAGAACCT | TACACCAGCTTTGGAGGAGCTGAA |
| *V2* | AGCAGATCCGTGATTACATGGCGA | TGCCTCTTCACTCTCTGCAACCAA |
| *23SrRNA* | TGTGGGCGTTAGAGCATTGAG | CACTTGGCTACCCAGCGTTTA |
| *AtpB* | TGAGAGGAATGGAAGTGATTGACA | TCAACAGGCTCCCCAAGAAC |
| *AtpE* | CGGTTCTGTGGAGCGGTTT | TGAGCTTCTTCCGGATCAATG |
| *16SrRNA* | CCGTTGGTGTTCTTTCCGAT | TTCAAGTCCGCCGTCAAATC |
| *rpl23* | GGATGGAATCAAATACGCAGTA | CGACCCAATGCTTTATTTCTG |
| Gene | Forward primer sequence (5’-3’) | Reverse primer sequence (5’-3’) |
| *rps7* | GCCAAAATCCATTCCAATTC | GGAGATGTACACGAGGAGATTG |
| *rps10* | CTGCCAACCAAGCGAAGAGT | ATCAATCGCTGGTGCGTTCT |
| *rps18* | CAACCTTTTCGCAAACCCAA | ATAATCAATTCGATCCCCCG |
| *rps19* | CGGGCATCTAGCATTCTACC | CCCAATTTGCGACCTACCATA |
| *rps20* | CACGCTCTTCTCCCTCTCCT | GTAGGAGGCGGACAGGCG |
| *Histone* | GGTCAACTTGTTGATTCCCCTCT | AACCGCAAAATCCAAAGAACG |
